# Lord’s Paradox in a Continuous Setting and a Regression Artifact in Numerical Cognition Research

**DOI:** 10.1371/journal.pone.0095949

**Published:** 2014-04-21

**Authors:** Kimmo Eriksson, Olle Häggström

**Affiliations:** 1 School of Education, Culture and Communication, Mälardalen University, Västerås, Sweden; 2 Centre for the Study of Cultural Evolution, Stockholm University, Stockholm, Sweden; 3 Mathematical Statistics, Chalmers University of Technology, Göteborg, Sweden; VU University Amsterdam, Netherlands

## Abstract

In this paper we review, and elaborate on, the literature on a regression artifact related to Lord’s paradox in a continuous setting. Specifically, the question is whether a continuous property of individuals predicts improvement from training between a pretest and a posttest. If the pretest score is included as a covariate, regression to the mean will lead to biased results if two critical conditions are satisfied: (1) the property is correlated with pretest scores and (2) pretest scores include random errors. We discuss how these conditions apply to the analysis in a published experimental study, the authors of which concluded that linearity of children’s estimations of numerical magnitudes predicts arithmetic learning from a training program. However, the two critical conditions were clearly met in that study. In a reanalysis we find that the bias in the method can fully account for the effect found in the original study. In other words, data are consistent with the null hypothesis that numerical magnitude estimations are unrelated to arithmetic learning.

## Introduction

Suppose that a researcher wants to study individual differences in how children respond to a training program. The predictor is a continuous measure of some individual property P. The dependent variable is improvement in ability, measured by a pre-training test and a post-training test. However, test scores are not perfect measures of ability. The same improvement in test scores from different pretest scores may therefore reflect different changes in ability. To control for this possibility the researcher may include the pretest score as a covariate in a regression analysis, thereby investigating whether property P predicts test score improvement *among children with equal pretest scores*. The topic of our paper is how this statistical adjustment may lead to incorrect conclusions because of regression artifacts arising from biased regression to the mean.

In 1999, Campbell and Kenny [1] devoted an entire book to warning about the dangers of statistical adjustments in comparisons of treatment effects between non-randomized groups. The basic problem was pointed out already thirty years earlier in a classic paper by Campbell and Erlebacher [2]. The problem has continued to attract attention, see [3] for a review of the literature and a novel analysis. One reason to write yet another paper on this topic is that the literature has focused on comparisons between groups. Although the logic is the same for analysis of a continuous individual property, this case deserves an explicit discussion. The direct motivation comes from a rather recent empirical study in numerical cognition [4], which analyzed the influence of a continuous property on learning. That study is particularly interesting because, in addition to a pre-training test and an end-of-training test, it included a follow-up test two weeks after the end of training with no training between end-of-training and follow-up. This feature will prove useful in assessing the size of the regression artifact.

### Formalizing the Problem

Let us formalize the abovementioned setup: A researcher wants to study individual differences in how children respond to a training program. The predictor is a continuous measure of some individual property P. The dependent variable is improvement in ability, (imperfectly) measured by a pre-training test score 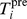 and a post-training test score 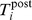. The researcher includes the pretest score as a covariate in a regression analysis. Denoting the property P level of child *i* by 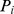, this means estimating the following regression model:
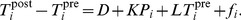
(1)


Here, *K* is the coefficient of interest, measuring the influence of property P on test score change. *D* is the intercept, *L* is a coefficient measuring the influence of pretest score on test score change, and the residual.

As discussed by other authors [3] it is irrelevant whether the dependent variable is test score change or simply the posttest score, because addition of the pretest score to both sides yields an equivalent model predicting posttest score instead of change:
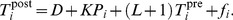
(1′)


Although inclusion of the pretest score as a covariate may seem both innocuous and sensible, it will lead to biased results when two critical conditions hold. The first condition is that P is correlated with pretest ability. The second condition is that test scores are not fully reliable measures of ability but subject to random within-individual variation, commonly represented by a “true score” model in which the test score is the sum of the child’s latent ability (true score) and a random error term of positive variance:
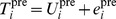
(2)




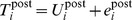
(3)


Improvement of test results will then reflect not only actual arithmetic learning (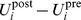) but also two random errors (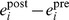).

### Why Regression Artifacts Arise

The only novelty of our setup is that property P is continuous. A classic setup is retrieved in the special case of P taking only values 0 or 1, indicating membership in one of two groups. Our first critical condition then reduces to the presence of a group differences in pretest scores. The risk of a regression artifact in that case was pointed out more than forty years ago by Campbell and Erlebacher [1]. The logic of their argument carries over directly to the continuous setting and goes as follows:

Children who are higher on property P will tend to have higher pretest scores than children who are lower on property P. By selecting to compare children with equal pretest scores, the researcher will inadvertently make a biased selection of the random errors. Specifically, consider a higher-P child and a lower-P child who happened to have the same pretest score. Equal test scores will arise by chance when a child with higher ability has less luck on the test than a child with lower ability. Because of regression to the mean, the child with worse luck on the first test–i.e., the one with higher ability–will tend to do better than the other child on the second test. Because children with higher P tend to have higher ability, equality in test scores will most often reflect a situation where the higher-P child has had worse luck and will therefore tend to be luckier on the next test. The observation that regression to the mean has this consequence of divergence between members of different groups is sometimes referred to as Kelley’s paradox [5]. The consequence is that, without any improvement in latent ability, the higher-P child is likely to score better on the next test than the lower-P child with the same pretest score. Any genuine changes in ability will be confounded by this bias in random errors.

Note how the bias was caused by the combination of initial differences and regression to the mean. The confounding effect on the results of the regression analysis is called a regression artifact.

### Outline of Paper

The paper consists of three studies. The first study is a mathematical analysis of the emergence of the regression artifact. We derive an unbiased estimator of the regression artifact under certain simplifying assumptions. The second study is a computer simulation to illustrate the regression artifact, leading up to a discussion of Lord’s paradox in a continuous setting. The third study applies our theoretical framework to a reanalysis of the main finding of the aforementioned study in numerical cognition [4].

## Study 1

### Analysis

To demonstrate how a regression artifact may arise in comparisons of groups, Campbell and Erlebacher [2] specified a model for test scores and abilities in two hypothetical groups. The model assumed abilities to be constant over time. Test scores could change, but only because of random errors. In the model it was therefore absolutely certain that finding any influence of group membership on change in test scores must be an artifact of the random errors.

Here we consider the case where children vary on a continuous property P rather than belong to one of two groups. We will demonstrate how a regression artifact arises when the test score difference is regressed on property P if the pretest score is included as a covariate. Adapting the model of Campbell and Erlebacher [2] to the case of a continuous property, we let child *i*’s actual ability (equal at pretest and posttest) be generated by the following equation:
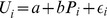
(4)


The first two terms specify a linear relationship between ability and property P, while the last term denotes unexplained between-individual variation in ability. We assume these random errors to be independently drawn from a normal distribution with mean 0 and standard deviation *s.*

Following equations (2) and (3), pretest and posttest scores of child *i* are obtained by addition of random errors to the child’s level of ability. We assume these random errors to be independently drawn from a normal distribution with mean 0 and standard deviation *σ.*

It is then possible to mathematically derive the expected size of the regression artifact. Under the model assumptions (2–4), the linear regression model (1) translates into
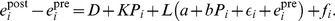



To analyze the results obtained from least-square estimation of this regression model we use the standard approach of letting the sample size tend to infinity, such that stochastic effects can be ignored. We can then identify coefficients between the left-hand and right-hand expressions to obtain
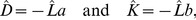
(5)and mean-square residual



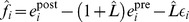
the law of large numbers. Least-square estimation entails minimizing this expression with respect to 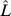. The minimum is attained for



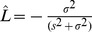
and plugging this into the second identity of equation (5) we obtain
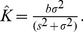
(6)


### Results and Discussion

For infinite samples, equation (6) gives the exact size of the regression artifact. For finite sized samples there will also be stochastic effects and (6) is then an unbiased estimate of the regression artifact. Note that this estimate is the product of the strength of the relation between property P and ability (*b*) and the proportion of the total variance in pretest scores that is accounted for by random within-individual variation. The regression artifact arises when these two entities are nonzero, which is equivalent to the two critical conditions stated earlier.

## Study 2

Following Campbell and Erlebacher [1], we will use computer simulations of our model (i.e., equations 2–4) to demonstrate the regression artifact arising from estimation of the regression model (1).

### Method

In order to simulate data we need to choose values for the model parameters *a, b, s,* and *σ.* We must also create a number of simulated children by assigning them a level of property P. To demonstrate the size of regression artifact that might arise in real empirical studies, we have chosen parameter values to roughly match the data of [4]. We will discuss that study in detail in a later section. For now it suffices to say that 105 children were measured on the linearity of their numerical magnitude estimations, corresponding to our property P. We used this dataset as our P levels, fixed throughout our simulations. Model parameter values *a = *0.5, *b = *0.4, *s = *0.1, and *σ = *0.1, were chosen to give a rough fit to the empirical data on test scores in [4]. Simulated abilities and test scores were then obtained from the model equations (2–4), with random terms obtained from a generator of random numbers built into Excel. One thousand simulated datasets were generated in this way. For each dataset we then estimated regression model (1).

### Results

**Figure 1:**
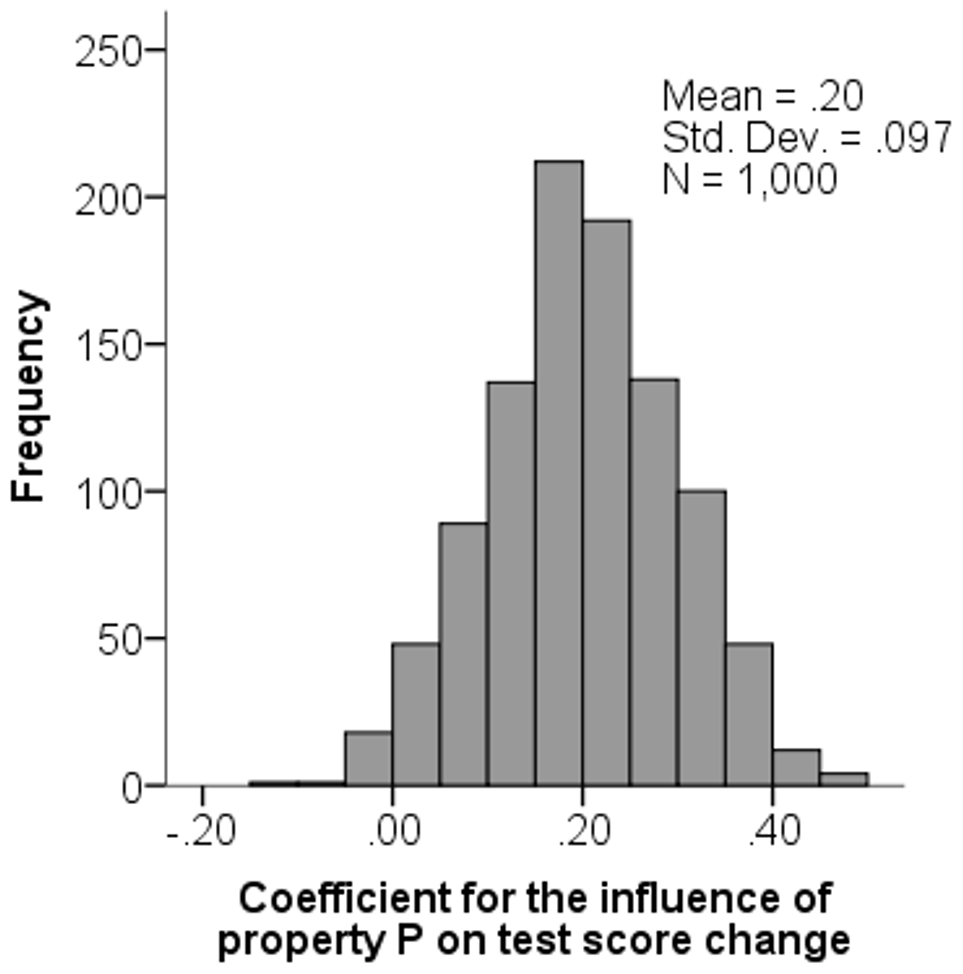
Histogram over the distribution of the unstandardized coefficient for the influence of property P on test score change, obtained from estimating regression model (1) in 1,000 simulated datasets in which actual abilities did not change between tests.

Our interest lies in 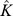, the estimated size of the influence of property P on test score change. Figure 1 shows how 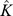 was distributed over the 1,000 simulations. It was greater than zero in more than 98 percent of simulations, with a mean value of 0.2004. Because of the large number of simulations, the mean value should be very close to the unbiased estimate given by equation (6). Indeed, plugging the parameter values (*b = *0.4, *s = *0.1, and *σ = *0.1) into equation (6) yields 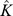 = 0.2.

Let us emphasize what these simulations tell us. They assume a situation where no child’s ability change between tests, so *a fortiori* there is no influence of property P on change in abilities. Despite this absence of a genuine effect, the simulations indicate that researchers using regression model (1) will almost always find a substantial influence of property P on change in test scores.

Note that the artifact tended to be half as large as *b*, the coefficient describing how property P relates to ability. Equation (6) explains why: The parameter values we used satisfied *s* = *σ*, which implies that random within-individual variation accounted for half the total variance in pretest scores, with the other half accounted for by between-individual variation in abilities.

### Discussion

**Figure 2:**
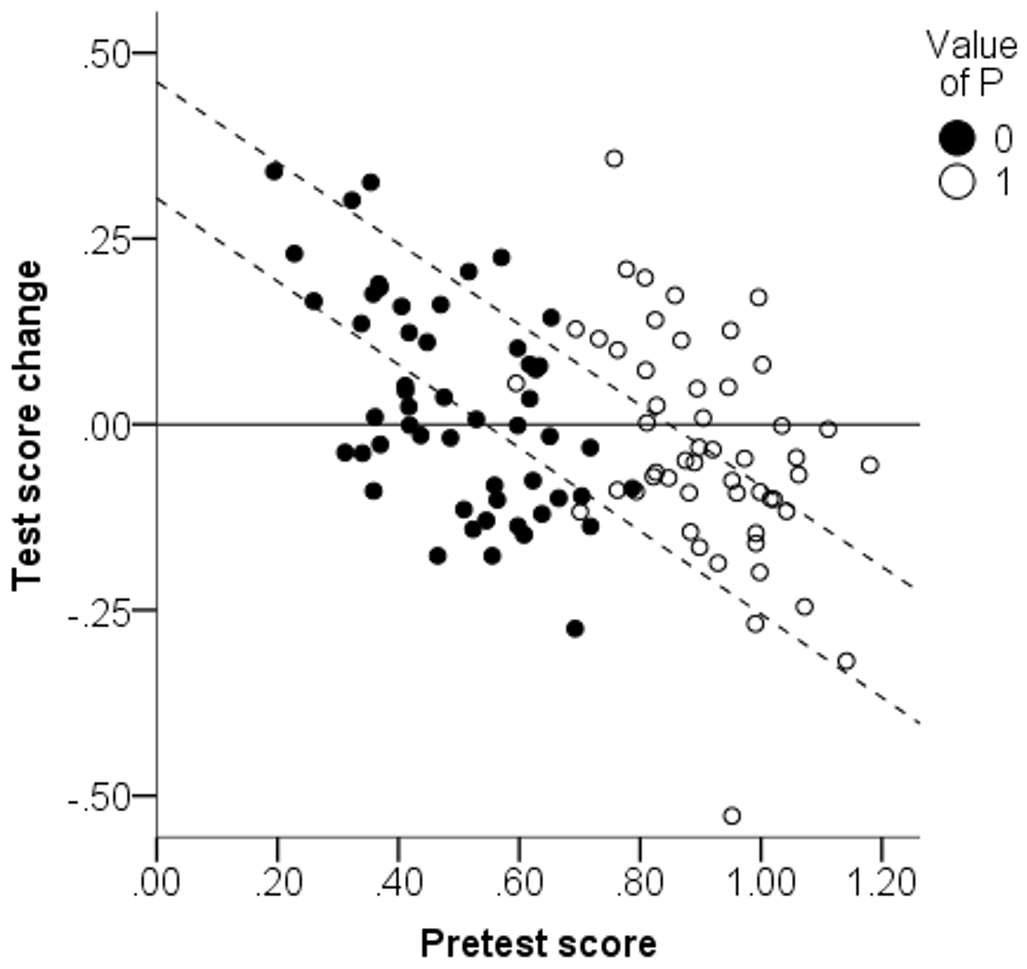
Scatter plot of simulated pretest score and test score change for children having either value 0 or 1 on property P. Latent abilities depended on P and did not change between tests.

We shall close the theoretical part of this paper by a discussion of Lord’s paradox. In its original form, Lord’s paradox is about comparison of groups. Let us therefore consider hypothetical children whose P values are either 0 or 1, such that groups can be based on P values. Using our simulation model we generated abilities and test scores for a hypothetical set of 105 children, equally distributed over the two P values. The results are presented in a scatter plot of pretest score against test score change (i.e., posttest minus pretest), see Figure 2. We shall discuss this plot in some detail.

First consider the solid line, indicating no change in test score. Because abilities did not change between tests in our model, the solid line is where all datapoints would have been if test scores had been perfect measures of ability. Because test scores included random errors in our model, the data points are instead distributed above and below the solid line to the same extent. Based on this observation an empirical researcher could draw the conclusion that *property P had no systematic influence on change in test scores*.

Now consider the dashed lines, showing the results of regressing test score change on pretest score in each group. These lines demonstrate another observation about this dataset: For children with the same pretest score, test score change tends to differ substantially between the high P group and the low P group. Based on this observation, an empirical researcher could instead draw the conclusion that *property P has a substantial systematic influence on change in test scores*.

This phenomenon, that the same data on pretests and posttests in two groups can yield conflicting conclusions depending on what aspect of the data is observed, was first pointed out in a classic paper by Lord [6]. It is commonly referred to as Lord’s paradox. By considering a continuous property P instead of group membership, we have the same paradox in a continuous setting.

So, which conclusion is correct? Given our knowledge about the model that generated this dataset, the answer is unambiguous: The first conclusion is correct and the second conclusion is incorrect. The relation between property P and change in test scores is just an artifact of regression to the mean and reflects no causal influence. Figure 2 allows an intuitive way to think about the regression artifact: Because of regression to the mean, high pretest scorers are more likely to score worse next time and data will therefore tend to slope downwards to the right. The high P group tended to score higher, and therefore lie to the right of the low P group. For data that slope downwards to the right, a slope that lies to the right of another slope will also look as if it lies above it.

The crux of the matter is that the empirical researcher would not know which model generated the data. Specifically, our mathematical analysis implies that equivalent data are generated by the following model:
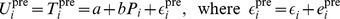





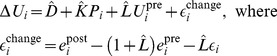
(7)


In words, this says that equivalent data would be observed if test scores were perfect measures of ability and if change in abilities were to some degree random but positively influenced by property P and negatively influenced by pretest ability. In such a world, the influence of P found by including pretest score as a covariate would be genuine and not a regression artifact. Instead it would be the analysis *without* the covariate that would point toward an incorrect conclusion, as it would not detect the positive influence of property P. Finally, in a world represented by a mixture of the two models both analyses would be biased.

The empirical researcher who wants to draw a conclusion about how much influence, if any, property P has on change in ability therefore needs additional knowledge about the underlying processes. Such knowledge may well exist. For instance, one may have an understanding about the mechanisms whereby ability changes. According to such understanding it might be implausible that ability would systematically decrease between tests among highly able children. This would support the first conclusion that the observed decrease in test scores among high pretest scorers is due to regression to the mean. It is also likely that a researcher has some knowledge about the extent of within-individual variation in test scores. Such knowledge can come from the nature of the test itself as well as from analysis of repeated tests with no treatment in-between. We shall later appeal to this kind of knowledge in our reanalysis of an empirical study.

Lord’s own version of the paradox presented data on weight change among male and female college students over an academic year [6]. The question was whether men and women tended to respond differently to the diet provided in the college dining halls. This is exactly the dichotomous version of the problem we study in this paper. Thus, individuals differ on a property P (gender in Lord’s problem), and the question is how this property of individuals affects their response to the same treatment. Most of the literature on Lord’s paradox focuses on another setup in which, depending on group membership, individuals either receive treatment or no treatment (control) and the question is if treatment has a different effect than no treatment. The statistical analyses look the same but interpretations will be different.

For a recent review and analysis of Lord’s paradox in treatment vs. control we refer to van Breukelen [3]. The focus of van Breukelen’s analysis is the circumstances under which the two methods of analysis (i.e., whether or not to include the pretest score as a covariate) will give unbiased results. Assuming non-randomized group assignment and presence of random errors, the conclusions can very briefly be summarized as follows: First, inclusion of the covariate will lead to biased results if groups differ in initial ability. This echoes what Campbell and Erlebacher [2] pointed out 40 years ago and is the same that we have said here in a continuous setting. Second, the alternative of not including the covariate may suffer from another source of bias, namely, that pretest ability actually influences the response to treatment, as in model (7). Third, both methods are unable to account for inherent differences between groups in how they respond to treatment, as treatment was only given to one of the groups. The third point obviously does not apply to the problem we (and Lord [6]) consider, in which everyone is given the same treatment.

To be able to test for the presence of bias in results, van Breukelen [3] suggests that experimental designs include two pretests with sufficient time in-between. If the analysis is unbiased, no effect should then be found treating the second pretest as posttest. The same logic applies if there are two posttests instead. In our below reanalysis of data from [4], we shall follow van Breukelen’s suggestion.

## Study 3

So far we have theoretically discussed why and when a certain statistical analysis method will produce a regression artifact. We now turn to an empirical study where this method of analysis was used. The background is an interesting and well-established research finding that children’s proficiency in solving arithmetic problems correlates with the linearity of their estimations of numerical magnitudes. Specifically, arithmetic performance tends to be better the more the child estimates numerical magnitudes in a linear rather than logarithmic way. This fact has been demonstrated in many studies, as reviewed by Booth and Siegler [4]. The objective of their 2008 study was to investigate whether arithmetic *learning* was also influenced by this property. The researchers’ hypothesis was that if children are trained on arithmetic problems, their arithmetic learning will be influenced by the linearity of their numerical magnitude estimations. Specifically, they predicted that children who make more linear estimations of numerical magnitudes would benefit more from training on arithmetic problems.

### Methods

Booth and Siegler [4] studied 105 first graders over several sessions across a few weeks, collecting a number of measures. For the purposes of our paper, only the below-mentioned measures are relevant as they were the ones that entered the critical analysis.

#### Numerical magnitude estimations

The first session included the task of estimating the positions of 26 different numbers (ranging between 2 and 98) on a number line between 0 and 100. The researchers then measured the linearity of a child’s estimations by calculating the proportion of variance in estimations explained by a best-fitting linear expression of the numbers to be estimated. This measure will be referred to as *R* ^2^_Lin_.

#### Test of arithmetic performance

A set of four arithmetic problems (9+18, 26+27, 17+29, and 49+43) was used to test arithmetic performance. Children were asked to solve these problems in the first session. A child’s performance on the problem set was measured as the average absolute error in answers divided by 100, referred to as “percent absolute error” (PAE). For instance, a child giving answers 28, 50, 50, and 80 to these problems would have made absolute errors 1, 3, 4, and 12, yielding an average absolute error of 5 and a PAE of 0.05.

In two subsequent sessions, children received training on the same problems. (Training occurred in four between-subject conditions using different instructional procedures. However, all conditions were pooled in the analysis of the main hypothesis. Because this is the analysis we are concerned with in the present paper, the fact that there were different conditions will not be relevant to our account.) At the end of training, children were again given the same problems to solve. In a follow-up session two weeks after the end of training, children solved the same set of problems for a third time. Thus, three performance measures were collected: pre-training (PAE_pre_), at end-of-training (PAE_end_), and at follow-up (PAE_followup_).

### Analysis

Booth and Siegler [4] analyzed their data using regressions that included pre-training performance (PAE_pre_) as a covariate. An impressive 39 percent of the variance in the test score difference PAE_end_−PAE_pre_ was explained by a multiple regression on *R* ^2^_Lin_ and PAE_pre_, with both factors coming out as highly significant predictors. A similar result was obtained for the difference in performance between the first session and the follow-up session: *R* ^2^_Lin_ and PAE_pre_ together explained 29 percent of the variance in PAE_followup_−PAE_pre_, again with both factors coming out as highly significant predictors. The researchers concluded that arithmetic learning is influenced by the linearity of children’s numerical magnitude estimations.

#### Assessing the risk of a regression artifact

Note that this study fits perfectly with our previous theoretical discussion. The researchers’ aim was to study how arithmetic learning is influenced by a certain continuous property, linearity of numerical magnitude estimations, operationalized by the quantity *R* ^2^_Lin_. This property is known to be related to arithmetic ability. The first condition for a regression artifact was therefore likely to be satisfied. Further, learning was measured as the change in test scores. These test scores measure how far off children were from the correct answers to difficult arithmetical problems. As a measure of arithmetic ability, this must be expected to suffer from substantial random errors. Children are likely to use guessing when they don’t know the right answer. They will then, by chance, sometimes come close to the right answer and sometimes not. Thus, the second critical condition for a regression artifact was also likely to be satisfied. Because the researchers used a method of statistical analysis that suffers from a regression artifact under the combination of these two critical conditions, we must expect their results to be biased. It might be that their finding was entirely due to the regression artifact. This calls for a reanalysis of their data.

#### Aim of reanalysis

Our aim is to estimate to what extent Booth and Siegler’s results suffer from the regression artifact and to assess whether or not their research conclusion still holds when the regression artifact is accounted for. To estimate the size of the regression artifact, we have conducted some additional analyses. We thank Julie Booth for sharing the raw data for this reanalysis. The data are presented in three scatter plots.

#### Relation between initial test score and R^2^_Lin_

**Figure 3:**
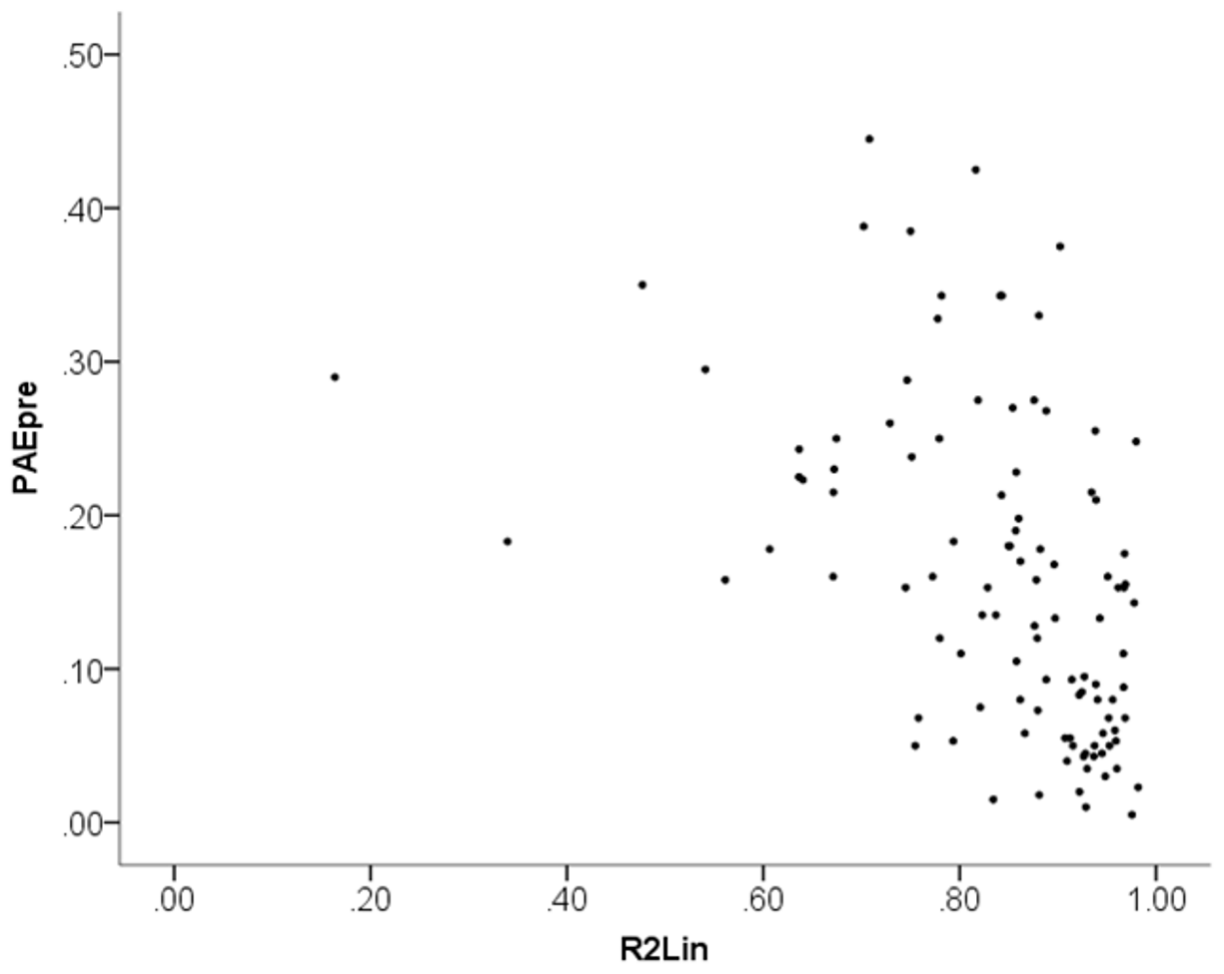
Scatter plot of pre-training performance (PAE_pre_) plotted against linearity of numerical magnitude representation (*R* ^2^_Lin_).

Figure 3 plots children’s pretest score (PAE_pre_) against their linearity of numerical magnitude estimations (*R* ^2^_Lin_). Recall that the test score measures percent absolute error in responses, so *lower values mean better performance*. It is clear from Figure 3 that children higher on *R* ^2^_Lin_ performed better on the pretest. This is the first of the two conditions that give rise to the regression artifact. A simple linear regression of PAE_pre_ on *R* ^2^_Lin_ yields an estimated value of −0.36 of the unstandardized coefficient. This corresponds to parameter *b* in our simulation earlier. However, it should be noted that the relation in Figure 3 seems to be non-linear, whereas the simulation model was linear.

#### Relation between test score change and R^2^_Lin_

**Figure 4:**
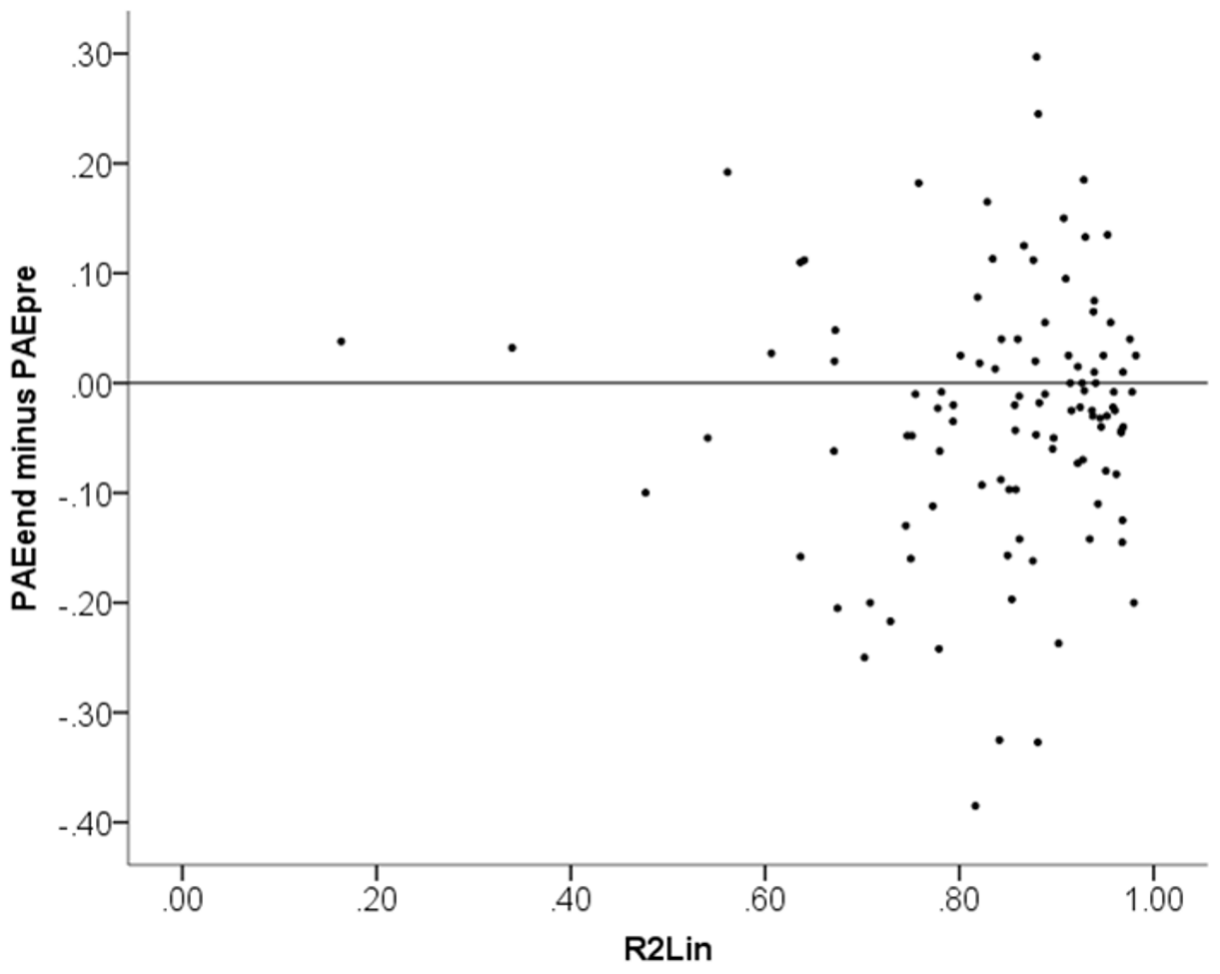
Scatter plot of change in performance from the first session to the end of training (PAE_end_−PAE_pre_) plotted against linearity of numerical magnitude representation (*R* ^2^_Lin_).

**Figure 5:**
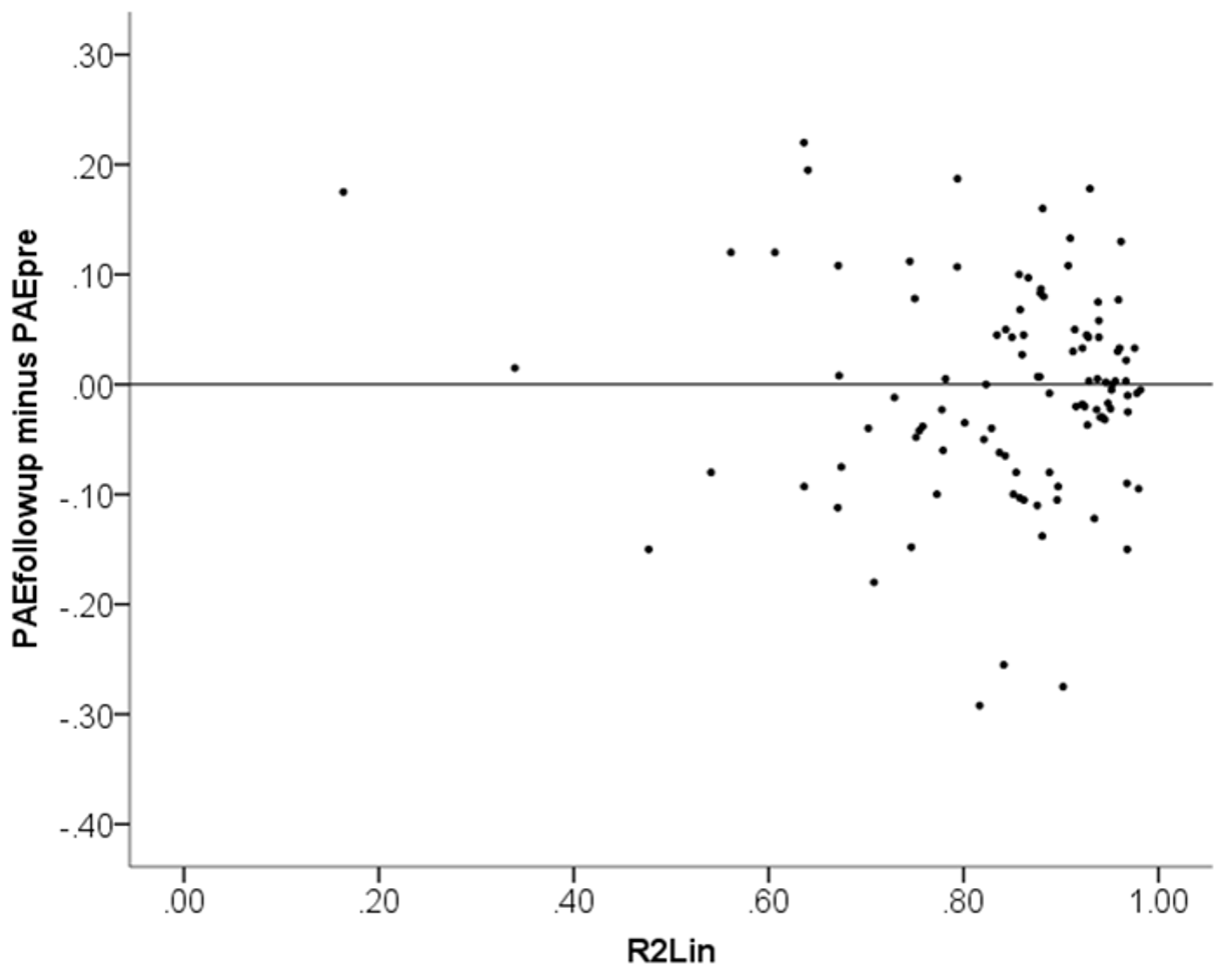
Scatter plot of change in performance from the first session to follow-up (PAE_followup_−PAE_pre_) plotted against linearity of numerical magnitude representation (*R* ^2^_Lin_).

Figure 4 plots the test score difference to the end-of-training test (PAE_end_−PAE_pre_) against *R* ^2^_Lin_. Similarly, Figure 5 plots the test score difference to the follow-up test (PAE_followup_−PAE_pre_) against *R* ^2^_Lin_. No correlations are evident in these plots. Statistical tests confirm that there was *no* statistically significant link between linearity of numerical magnitude estimations (*R* ^2^_Lin_) and improvement in test scores from training, neither when measured at end of training (PAE_end_−PAE_pre_), *r*_S_ *= *.07, *p = *.47, nor when measured at follow-up (PAE_followup_−PAE_pre_), *r*_S_ *= *.03, *p = *.76. To avoid any misunderstanding of this result, let us point out that the meaning of the positive sign of the non-significant correlations is that linearity of numerical magnitude estimations *negatively* correlated with test score improvement. In other words, this analysis if anything suggests an effect in the opposite direction to Booth and Siegler’s reported finding. Next we replicate Booth and Siegler’s analysis. Including the initial test score (PAE_pre_) as a covariate in a linear regression of the test score differences on *R* ^2^_Lin_, we find estimated unstandardized coefficients of −0.27 at end of training and −0.26 at the follow-up session. These values correspond to the outcome variable in our simulations in Study 2 (although with the opposite sign because PAE test scores measure negative ability). However, note that the real data do not support that relations between variables are linear, which they were assumed to be in our simulations.

#### Lord’s paradox in a continuous setting

Taken together, the above analyses show that the dataset exhibits our continuous version of Lord’s paradox. On the one hand, simple correlations indicate that linearity of numerical magnitude estimations does not have any positive influence on test score improvement. On the other hand, when the pretest score was included as covariate the results clearly indicate a positive influence on test score improvement. Which of these results best reflect the answer to the real research question – whether there is any influence on *arithmetic learning*? Recall that we have very good reason to believe that test scores include substantial random variation due to guessing. When a child happens to make worse guesses on the second test than on the first test it is incorrect to infer that the child’s arithmetic ability has deteriorated. Similarly, it is incorrect to infer that a child’s arithmetic ability has improved if a child happens to make better guesses on the second test than on the first test. For this reason, it is crucial to estimate how much of children’s changes in test scores are due to learning and how much is due to random variation.

#### Arithmetic learning

**Figure 6:**
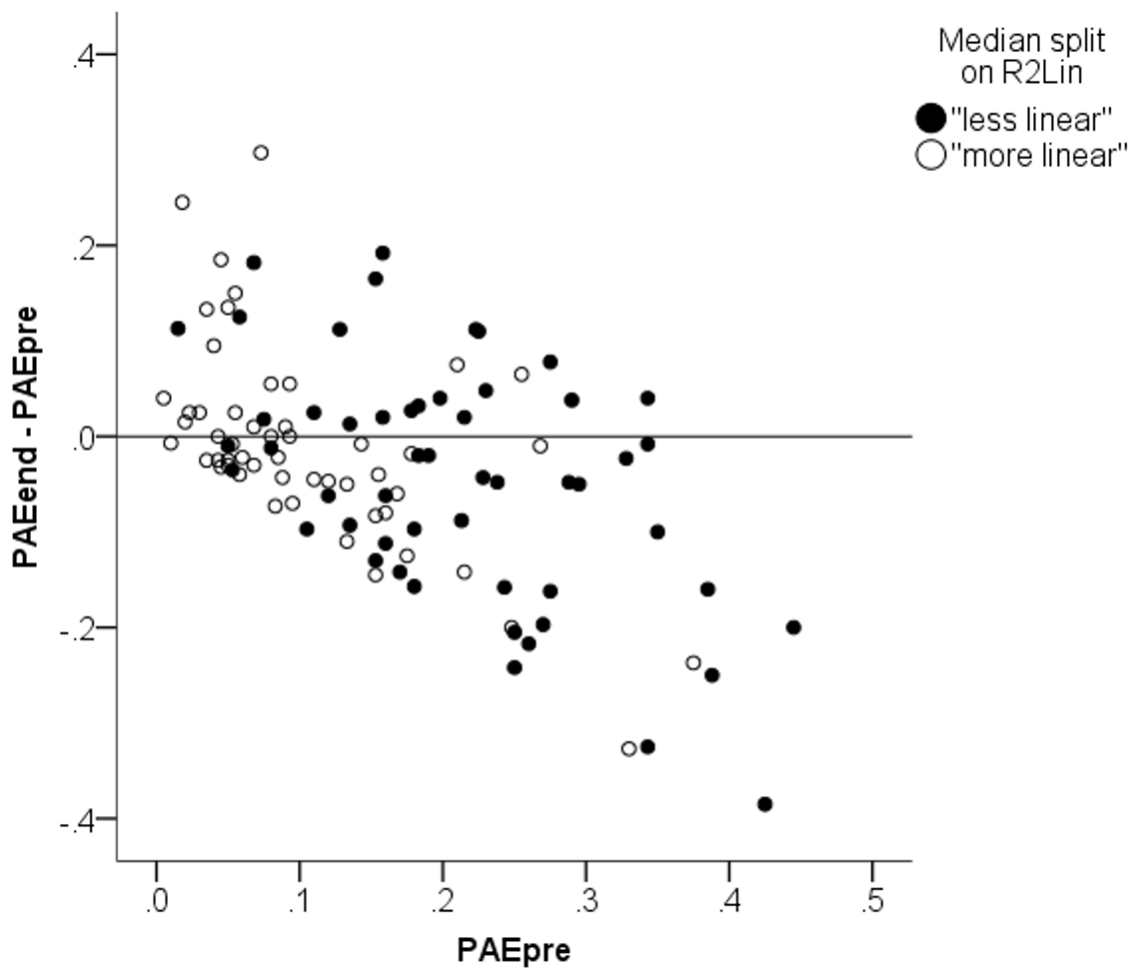
Scatter plot of change in performance from the first session to end of training (PAE_end_−PAE_pre_) plotted against initial test score. Black and white dots signify “more linear” and “less linear” children according to a median split based on *R* ^2^_Lin_.

Did arithmetic learning take place at all? In Figures 3 and 4 it is not obvious to the eye that later test scores showed any systematic improvement from the pretest. A statistical analysis reveals that the median change in test score from pre-training to end of training was small (0.022, less than a fifth of the standard deviation of 0.118) but statistically significant, *p = *.014, signed rank test. From pre-training to follow-up, however, there was no statistically significant change in scores (median = 0.005), *p = *.72, signed rank test. This indicates that arithmetic learning was weak or even non-existent. Changes in test scores seem mainly to be driven by random variation. We must therefore expect biased regression to the mean to be a strong driver of the results of Booth and Siegler’s analysis. Figure 6 provides another way of looking at the data. Here we plot test score change (to the end-of-training session) against the pretest score. To illustrate *R* ^2^_Lin_ in the same plot we have conducted a median split of children into “more linear” and “less linear” (i.e., above and below median on *R* ^2^_Lin_, respectively). Figure 6 is analogous to how we presented Lord’s paradox in simulated data in Figure 2. In the plot it is apparent that children who performed well on the pretest tended to exhibit a worsening of performance on the next test. To reconcile this pattern with the hypothesis that test scores differences reflect actual learning, we must accept that children with high ability tend to lose ability from arithmetic training. This seems implausible. Indeed, Booth and Siegler’s theoretical argument clearly assumes that training increases ability.

#### The regression artifact in a no-training period

Our final analysis capitalizes on Booth and Siegler’s inclusion of a follow-up test. Between the tests at end-of-training and follow-up no child received training. This is as close as an empirical study can get to ascertain that no actual learning affects the results between two tests. As suggested by van Breukelen [3], we obtain an estimate of the size of the regression artifact by running the same statistical method using the first posttest as a pretest to the second posttest. In other words, we regress the test score difference PAE_followup_−PAE_end_ on *R* ^2^_Lin_, including PAE_end_ as a covariate. The result is an estimated unstandardized coefficient of −0.25. This result is essentially identical to the results of our replication of Booth and Siegler’s analyses, which yielded unstandardized coefficients of −0.27 and −0.26. We conclude that there is no evidence of any influence of *R* ^2^_Lin_ on test score changes beyond the influence that stems from random variation in test scores.

## Discussion

In this paper we have discussed a pitfall in regression analysis of individual differences in change of test scores between a pretest and a posttest. Inclusion of the pretest score as a covariate may produce a regression artifact of a kind that has been discussed for a long time in the statistical literature [1]–[3], [5]–[6]. The problem arises when two critical conditions hold, namely, when the property used to predict learning is correlated with initial ability and when individuals’ test scores show some random change between tests even if their actual ability does not change. Under the second condition, test scores on the second test will exhibit regression to the mean. Under the first condition, this will make individuals that happen to have the same pretest score tend to differ systematically on the second test. The effect is that inclusion of the pretest score as a covariate in the regression will make it look as if the property actually predicts learning, when it actually just predicts the pretest score. Whereas previous literature has focused on the case when the property is group membership, we have discussed how the same problem occurs in a continuous setting.

In an empirical study, Booth and Siegler [4] included the pretest score as a covariate in their regression analyses of how arithmetic learning is influenced by the linearity of numerical magnitude estimations. Their study satisfied the conditions under which the regression artifact arises and their results were therefore likely to be biased. Reanalysis showed that their data are consistent with the null hypothesis that arithmetic learning is not influenced by the linearity of numerical magnitude estimations. In other words, the null hypothesis should not be rejected. Thus, the regression artifact made the researchers draw the wrong conclusion about their research question. We first pointed out the regression artifact to the journal that published the original study. They responded that they receive far more manuscripts than they can publish and therefore declined to publish a refutation. We would like to offer an alternative viewpoint: Readers have reason to believe that publication in a highly selective journal is a reliable sign of correctness. Journals would safeguard this reliability by making sure to inform their readers in all (hopefully few) cases where the conclusions of research they have published later turns out to be seriously flawed. Such a practice would also help to avoid prolonged popular belief in incorrect findings [7].
